# Establishing and Optimizing Kangaroo Mother Care Practices Among Very-Low-Birth-Weight Neonates in a Tertiary Neonatal Intensive Care Unit (NICU): A Quality Improvement Approach

**DOI:** 10.7759/cureus.113558

**Published:** 2026-07-28

**Authors:** Purnima Sahoo, Niyati Das, Santosh K Panda

**Affiliations:** 1 Child Health Nursing/Newborn, Kalinga Institute of Nursing Sciences, Kalinga Institute of Industrial Technology (KIIT) Deemed to be University, Bhubaneswar, IND; 2 Paediatrics, Kalinga Institute of Nursing Sciences, Kalinga Institute of Industrial Technology (KIIT) Deemed to be University, Bhubaneswar, IND; 3 Pediatrics, Kalinga Institute of Medical Sciences, Kalinga Institute of Industrial Technology (KIIT) Deemed to be University, Bhubaneshwar, IND

**Keywords:** intensive care units, kangaroo mother care, premature infant, skin-to-skin contact, very low birth weight

## Abstract

Background

Kangaroo Mother Care (KMC) is an evidence-based practice that improves outcomes in very-low-birth-weight (VLBW) neonates by lowering neonatal mortality, hypothermia, sepsis, and hospital stay duration while enhancing breastfeeding, physiological stability, and neurodevelopmental outcomes. Despite recommendations for initiating KMC within the early postnatal period (within the first 24-72 hours of life) and maintaining prolonged (six to eight hours/day) daily skin-to-skin contact, implementation and continuation of KMC remain inconsistent in neonatal intensive care units (NICUs). This study aims to improve early initiation within seven days of birth and duration of KMC among VLBW neonates in the NICU.

Methodology

The implementation phase was conducted from September to December 2024 in the NICU of Kalinga Institute of Medical Sciences, Kalinga Institute of Industrial Technology Deemed to be University, Bhubaneswar, India. This quality improvement (QI) study aimed to improve the early initiation and duration of KMC among haemodynamically stable VLBW neonates weighing <1.5 kg. The QI team comprised the principal investigator, a neonatologist, a senior resident, two senior nursing officers, and NICU staff. Interventions were implemented through multiple Plan-Do-Study-Act (PDSA) cycles. During the planning phase, baseline gaps in KMC practices were identified through team discussions. The implementation phase included staff training on KMC eligibility, positioning, monitoring, and safe practices, alongside enhanced maternal counselling using bedside demonstrations and audiovisual aids. The study phase evaluated intervention effectiveness using predefined indicators and stakeholder feedback. In the act phase, successful strategies were standardized and refined for subsequent PDSA cycles, ensuring sustained improvement in KMC implementation.

Results

With the implementation of the QI interventions, the median time to initiation of KMC decreased from 18 days at baseline to 10 days during the first PDSA cycle and further to seven days during the second cycle, indicating substantial improvement in early KMC initiation among VLBW neonates. The median daily duration of KMC increased progressively from 3.28 hours at baseline to 4.08 hours in the first cycle and 6.19 hours in the second cycle, demonstrating improved adherence to prolonged KMC practices. In the present study, maternal age, gestational age, and mode of delivery differed significantly across baseline, PDSA1, and PDSA2 phases, suggesting potential case-mix variation over time.

Conclusion

QI interventions led to a marked improvement in both the early initiation and the duration of KMC among VLBW neonates. Key gaps were successfully identified in the initial phases. Structured QI interventions effectively improved the early initiation and daily duration of KMC among VLBW neonates in the NICU. Identification of practice gaps, followed by targeted interventions including staff training, caregiver education, standardized KMC protocols, and regular monitoring through PDSA cycles, enhanced adherence to KMC practices and promoted more consistent implementation of KMC in the NICU.

## Introduction

Kangaroo Mother Care (KMC) is a cost-effective, evidence-based intervention recommended for preterm and very low birth weight (VLBW) neonates to improve survival and neonatal outcomes [1,2]. Early initiation of KMC refers to the commencement of skin-to-skin contact within the early postnatal period, preferably as soon as the neonate becomes clinically stable, while optimal duration refers to prolonged (six to eight hours per day) and continuous daily skin-to-skin care to achieve maximum physiological and developmental benefits [3].

Early initiation and optimal duration of KMC are associated with improved thermoregulation, cardiorespiratory stability, breastfeeding, weight gain, sleep organisation, neurodevelopment, parent-infant bonding, and reduced risks of sepsis, hypothermia, and neonatal mortality [1,4,5]. Furthermore, successful and sustained KMC depends on several interrelated factors, including neonatal stability, trained healthcare staff, maternal knowledge and confidence, family support, institutional policies, adequate infrastructure, NICU staffing and workload, and effective monitoring systems to ensure infant safety during KMC [6,7]. Despite global recommendations, KMC implementation in many NICUs remains suboptimal due to delayed identification of eligible neonates, inadequate staff training, lack of standardised guidelines, maternal anxiety and fatigue, limited privacy and space, insufficient counselling, and increased workload among healthcare providers for early initiation of KMC [7-9]. These barriers often result in delayed initiation and reduced duration of KMC, which may negatively affect neonatal recovery, prolong hospital stay, decrease exclusive breastfeeding rates, and limit the overall effectiveness of KMC practices in improving neonatal outcomes [4,5,9].

Emerging evidence highlights the importance of early initiation of KMC, including within the first 24 hours of life in clinically stable preterm infants under close monitoring. Early initiation is associated with improved weight gain, faster recovery, and shorter hospital stays compared to delayed initiation [10-15]. Despite these advantages, the implementation of early and sustained KMC remains inconsistent in many neonatal intensive care units (NICUs), particularly in low- and middle-income countries. Key barriers include inadequate infrastructure, insufficient staff training, high workload, sociocultural factors, and concerns regarding the safety of unstable neonates [16]. Addressing these gaps requires the integration of KMC in routine NICU care through structured, system-based strategies.

In this context, the present study aimed to enhance early initiation within seven days and increase the daily duration of KMC among VLBW neonates admitted to the NICU using a structured quality improvement (QI) approach, intending to improve adherence to recommended practices and achieve better neonatal outcomes. The World Health Organization (WHO) recommends at least eight hours of daily KMC; achieving this target in resource-limited settings remains challenging. Prior to this QI initiative, KMC was routinely encouraged and practised in our tertiary NICU. However, opportunities existed to further strengthen the consistency, monitoring, and duration of KMC practices. Baseline assessment identified scope for earlier initiation and prolonged daily KMC among eligible VLBW neonates. Hence, this QI initiative was designed to establish and optimize KMC practices through structured monitoring, staff engagement, and system-based interventions integrated into routine neonatal care.

## Materials and methods

This prospective QI research study is a part of the main research study that took place in the NICU of a tertiary medical centre, located in the state of Odisha. The research protocol was equally reviewed and approved by the local Institutional Review Board (IRB) and Ethics Committee Kalinga Institute of Medical Sciences (KIMS), KIIT Deemed to be University, Bhubaneswar, Odisha, following approval number KIIT/KIMS/IEC/1867/2024 on June 25, 2024, the federal IRB through the Clinical Trial Registry of India (CTRI - registration #CTRI/2024/09/074128, https://ctri.nic.in/Clinicaltrials/login.php?rec=1). The study was accomplished over a three-month period (September 15, 2024, to December 15, 2024), with the objective of implementing early KMC and improving the duration of KMC among all haemodynamically stable VLBW neonates (<1.5 kg) who were not receiving invasive ventilation or central line catheters, experiencing shock, having seizures, or receiving phototherapy.

Following confirmation of eligibility, written informed consent was obtained from the mother prior to enrolment of the mother-infant dyad in the study. A total of 60 mother-infant dyads participated, comprising haemodynamically stable VLBW neonates and mothers willing to participate in KMC. In cases of multiple births, an additional caregiver was involved to facilitate KMC and maintain adequate duration of skin-to-skin contact for each neonate. This strategy was implemented to address practical difficulties in providing continuous KMC to more than one infant simultaneously and to enhance adherence to prolonged KMC practices in the NICU setting. To improve early KMC within seven days, the daily duration of KMC practices in the NICU, a multidisciplinary QI team was constituted. The team comprised the principal investigator, a neonatologist, a senior resident in neonatology, two senior nursing officers, and the nursing in-charge, who collaboratively planned, implemented, supervised, and monitored the QI interventions. The responsibilities of the team included identifying barriers to KMC implementation, training healthcare staff and caregivers, standardizing KMC practices, and reviewing progress throughout the study period. Baseline data were collected prior to the initiation of interventions to assess the existing KMC practices in the NICU. The number of dyads in baseline 13 (PDSA 1) is 24, and in PDSA 2, it is 23. These data included the time (in days after birth) at which KMC was first initiated for eligible VLBW neonates and the average daily duration (in hours per day) of KMC provided before implementation of the QI strategies. The baseline assessment helped in identifying gaps in practice and served as the reference point for evaluating the effectiveness of subsequent interventions implemented through the PDSA cycles. After the baseline data collection during the first two weeks of the study, the QI team conducted in-depth interviews with parents and caregivers of eligible VLBW neonates about existing KMC practices before implementing the intervention strategies.

These interviews were carried out during the pre-intervention phase to identify barriers that limited the timely initiation and continuation of KMC in the NICU. Parents' interactions were held for about one week to ask about their knowledge, perceptions, concerns, and practical difficulties related to KMC practice.

Commonly identified limiting factors, including fear of handling small babies; maternal fatigue and pain; lack of awareness regarding the benefits of KMC and anxiety about the infant’s clinical condition; inadequate family support, mainly the mother having a C-section delivery, are difficulties in maintaining prolonged skin-to-skin contact within the NICU environment. The information obtained from these interviews was used to design targeted interventions, including staff training, caregiver education, counselling sessions, and structured monitoring strategies, during subsequent PDSA cycles. In an effort to assess caregiver (mother and father) and nursing staff awareness and barriers related to KMC, both caregivers and nursing staff were included in qualitative focus groups to identify barriers to KMC. The QI team then utilized fishbone analysis (Ishikawa diagram) to identify the major barriers to initiating and maintaining KMC with both nursing staff and parents. In the fishbone analysis, the identified barriers were prioritized based on their perceived impact on the problem, feasibility of implementation, and alignment with available resources. These findings directly informed the design of the interventions tested in each PDSA cycle and were structured to test changes that specifically addressed the underlying causes identified through the fishbone exercise, ensuring that improvement efforts were focused, evidence-informed, and aligned with the key drivers of the problem. The primary barriers identified included lack of awareness, fear of handling a VLBW infant, inadequate support, and low nurse motivation.

As part of the QI project, weekly meetings of the QI team were held throughout the study period to review the progress of KMC implementation, assess compliance with the planned interventions, and identify ongoing challenges in practice. During these meetings, findings from focus group discussions involving healthcare staff and caregivers were reviewed to identify barriers to the early initiation and optimal duration of KMC. Key barriers identified included poor awareness of KMC protocols, inconsistent counselling approaches, high staff workload, maternal discomfort, and inadequate involvement of family members. Based on these findings, practical and context-appropriate interventions were formulated and evaluated through sequential Plan-Do-Study-Act (PDSA) cycles. Mainly, in these two PDSA cycles, four phases are present. During the planning phase, targeted strategies such as staff training programs, caregiver counselling sessions, standardized KMC recording charts, and reminder systems were developed. In the implementation phase, these interventions were introduced in the NICU. The study phase involved assessing their impact by analyzing data on KMC initiation time and daily duration. In the action phase, effective interventions were sustained, while less effective measures were refined before being incorporated into subsequent cycles. This continuous iterative approach enabled regular monitoring and gradual enhancement of KMC practices in the NICU.

Intervention

The PDSA cycles are listed below (Table 1).

**Table 1:** Plan of action in the Plan-Do-Study-Act (PDSA) cycle

PDSA cycle	Plan	Do	Study	Act
1	Educate and sensitize staff/parents, bridge communication gaps	Bedside demonstration, audiovisual materials, provide specific gowns/sheets	Median KMC increased to 4.08 hours/day.	Interventions standardized and sustained into daily clinical workflow.
2	Introduce night KMC, identify KMC champions, behavioural reinforcement tracking	Token distributions, smiley face colour codes, nighttime tracking cards	Median KMC increased to 6.19 hours/day.	Interventions adopted permanently; institutionalized into routine rounds.

Interventions were executed in succession to accomplish the KMC-related modifications suggested by the research team. Regular team meetings were held to assess the implementation process, track progress, and recognize and tackle any new challenges or obstacles that might hinder the effective implementation of KMC.

Study and evaluation of intervention

Process indicators included adherence to the nurses’ education programme during the first PDSA cycle and compliance with prescribed practices by resident neonatologists during the second PDSA cycle. The outcome indicator was the median duration of KMC practised across the different phases of the study, namely, PDSA1 and PDSA2. The QI team reviewed the run chart on a weekly basis and displayed it on the notice board to enhance awareness among healthcare workers and caregivers.

Study design and setting

This was a prospective QI study conducted in the NICU of a tertiary care teaching hospital in Odisha, India, over a three-month period, from September 15, 2024, to December 15, 2024. The study aimed to improve the early initiation and duration of KMC among VLBW neonates.

Study population

The study population comprised neonates with birth weights of less than 1,500 grams who were haemodynamically stable and not requiring invasive ventilation. Neonates were included if they were clinically stable and eligible for routine neonatal care and assessment. Neonates requiring invasive ventilation or central venous catheters were excluded from the study. In addition, neonates with shock, seizures, critical clinical instability, those receiving phototherapy at the time of assessment, and neonates with major congenital anomalies were also excluded from participation.

Eligible mother-infant dyads were enrolled after obtaining written informed consent from the mother or primary caregiver. In cases of multiple births, additional caregivers were included as part of the caregiving unit.

QI team and baseline assessment

A multidisciplinary QI team was constituted, comprising the principal investigator, neonatologist, senior resident, and nursing officers. Baseline data on timing of initiation and duration of KMC were collected. To identify barriers to KMC implementation, structured interviews and discussions were conducted with caregivers and nursing officers. A fishbone analysis was performed to categorize barriers, which included lack of awareness, fear of handling VLBW infants, inadequate support, and low staff motivation.

Run chart analysis

The run chart demonstrated a sustained reduction in the median age of KMC initiation and a progressive increase in daily KMC duration following successive PDSA cycles, indicating effective integration of KMC practices into routine NICU care. Interventions were implemented through two sequential PDSA cycles, where PDSA Cycle 1 focused on improving awareness and knowledge through staff training, maternal education, counselling sessions, and provision of KMC-friendly resources, such as gowns. PDSA Cycle 2 focused on sustaining and enhancing KMC practices through identification of KMC champions, introduction of night KMC, establishment of a breast milk expression room, and use of tracking tools to monitor KMC duration. Weekly QI meetings were conducted to review progress and modify interventions based on observed outcomes.

Outcome measures

The primary outcome focuses on the time to initiate KMC (in days after birth), while the secondary outcome focuses on the average daily duration of KMC (in hours per day). During data collection and monitoring, maternal and neonatal demographic and clinical characteristics were systematically recorded in a Microsoft Excel database (Microsoft Corporation, USA) prior to analysis. Maternal variables included age, parity, mode of delivery, and educational status, while neonatal variables included gestational age, birth weight, sex, clinical stability, and age at initiation of KMC. The collected data were checked for completeness and accuracy before statistical analysis. Statistical analysis was performed using IBM SPSS Statistics for Windows, version 20.0 (IBM Corp., Armonk, NY, USA). Continuous variables, including the timing of KMC initiation and daily duration of KMC, were summarised using the median and interquartile range due to the non-normal distribution of data, whereas categorical variables were presented as frequencies and percentages. The findings were used to compare changes in KMC practices across the baseline period and subsequent PDSA cycles.

Statistical analysis

Data were analysed using SPSS version 20.0 (SPSS Inc., Chicago, IL, USA). Continuous variables were expressed as median, as appropriate. Categorical variables were presented as frequencies and percentages. Run charts were used to illustrate trends in KMC initiation and duration across the study phases.

## Results

**Figure 1:**
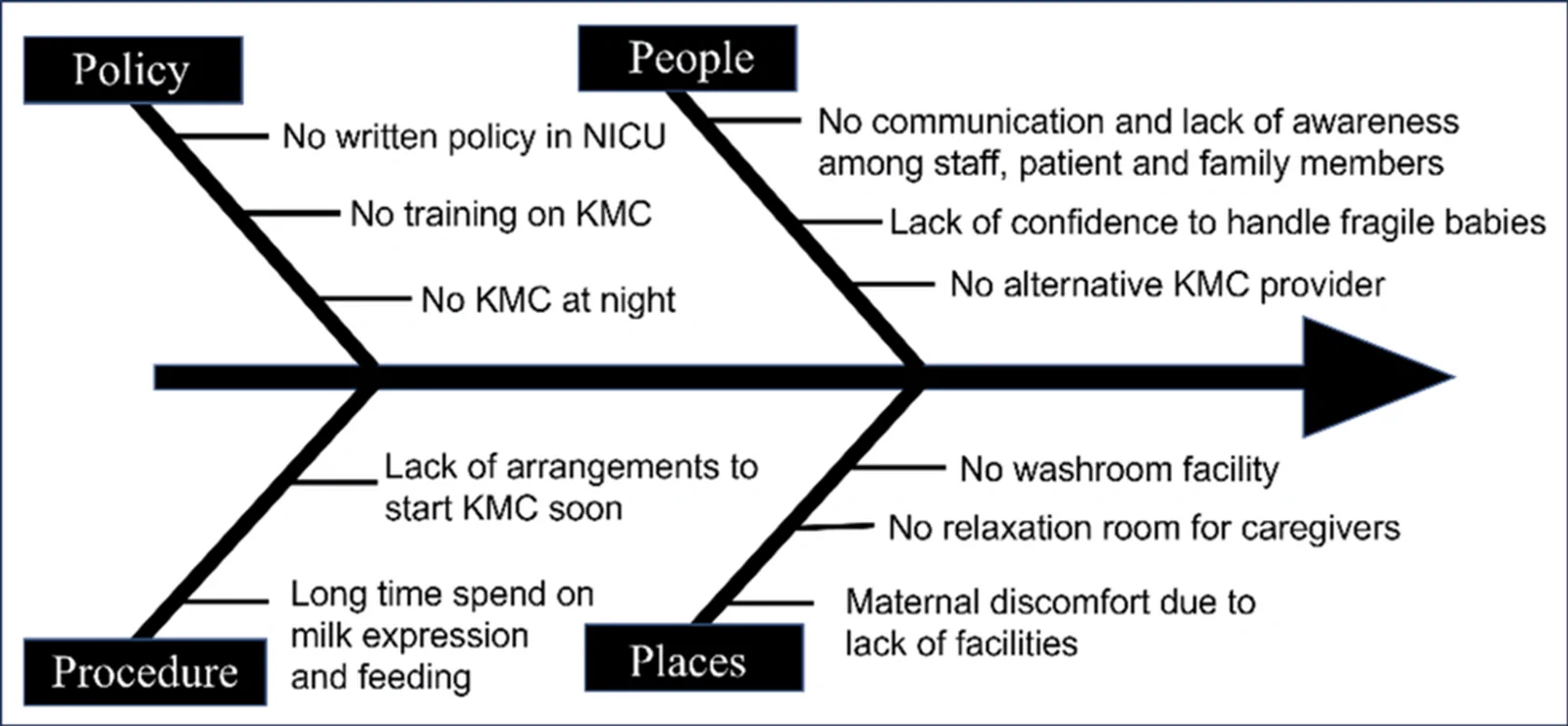
Fish bone analysis Fishbone analysis of barriers to Kangaroo Mother Care (KMC) implementation in the NICU. FBA was conducted as a root cause analysis tool to identify factors contributing to delayed initiation and inadequate duration of KMC in the NICU. Identified causes were categorized into four domains: policy, people, procedure, and place.

Fishbone analysis identified multiple barriers to optimal KMC implementation, which were categorized into four domains: policy, people, procedure, and place (Figure 1).

Policy-related barriers included the absence of a written KMC policy, lack of formal staff training, and non-availability of night-time KMC. People-related barriers comprised inadequate awareness and communication among healthcare staff and families, maternal lack of confidence in handling fragile neonates, and the absence of alternative caregivers. Procedure-related barriers included delays in KMC initiation and prolonged time required for breast milk expression and feeding. Place-related barriers involved inadequate washroom facilities, lack of caregiver rest areas, and poor NICU infrastructure affecting maternal comfort. These findings informed the design of subsequent PDSA cycles aimed at addressing the identified barriers and improving KMC implementation.

**Figure 2:**
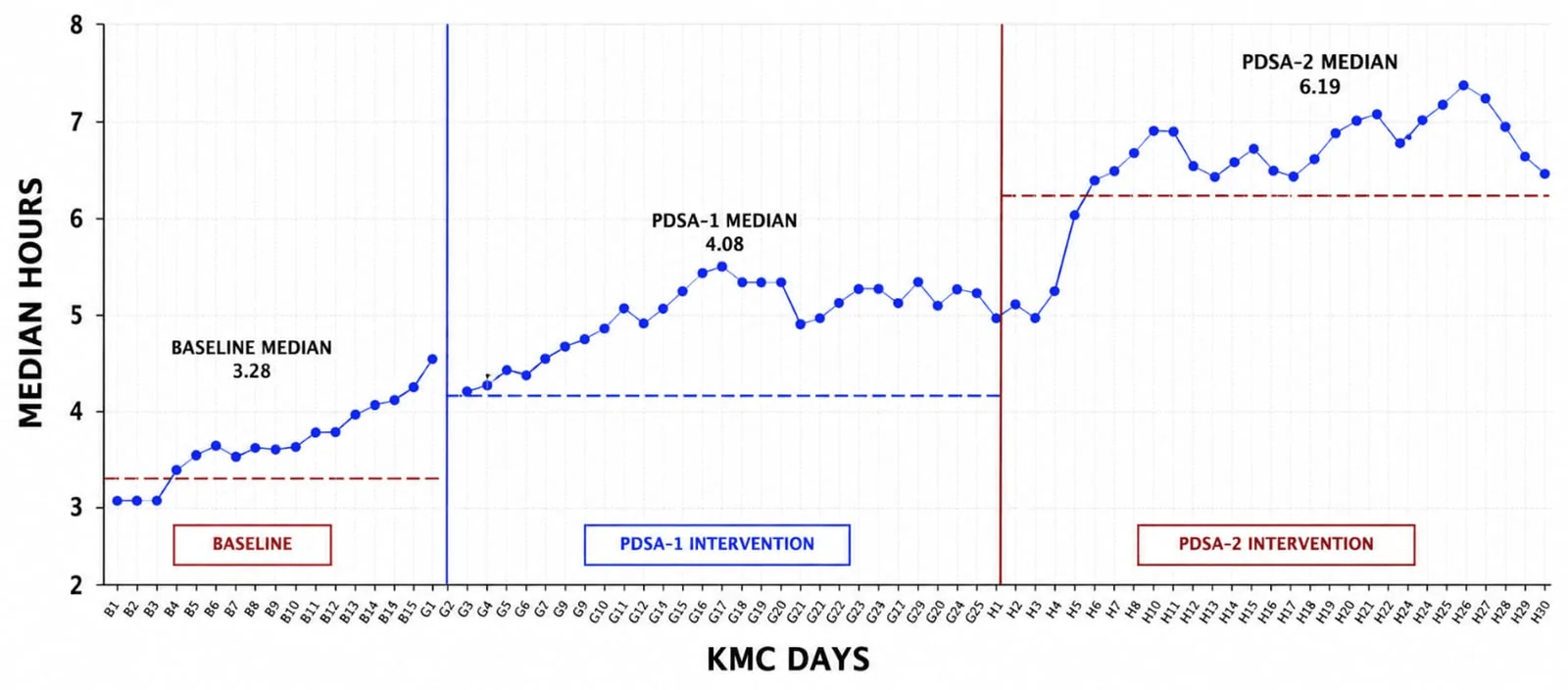
Run chart showing median hours and Kangaroo Mother Care (KMC) days during baseline and Plan-Do-Study-Act (PDSA) 1 and PDSA 2

The PDSA analysis demonstrated that a structured QI approach resulted in substantial improvements in KMC implementation (Figure 2). The median time to KMC initiation improved progressively across the improvement cycles, decreasing from 18 hours at baseline to 10 hours during PDSA Cycle 1 and further to seven hours in PDSA Cycle 2. Similarly, the average daily duration of KMC increased from 3.28 hours at baseline to 6.19 hours by the end of the second PDSA cycle. These findings indicate improved adherence to KMC protocols by healthcare providers and greater participation by caregivers. The improvements observed were not limited to quantitative outcomes but also reflected a transition in KMC practice from being irregular and inconsistent to becoming a routine component of neonatal care. Enhanced staff engagement, increased caregiver involvement, and improved consistency in KMC delivery suggest that both organizational and behavioural changes were successfully integrated into clinical practice, supporting the sustainability of the intervention.

A major contributor to these improvements was the implementation of multiple complementary interventions across successive PDSA cycles. Educational sessions, supported by tailored presentations and validated instructional videos, improved the knowledge and confidence of healthcare workers and parents, thereby promoting standardized KMC practices. Daily counselling reinforced these messages and encouraged continuous parental participation. Operational changes, including the provision of designated KMC gowns and structured support for mothers, facilitated routine implementation within the neonatal unit. Behavioural interventions also played an important role. The introduction of a KMC monitoring card with a simple smiley-face indicator provided regular feedback, promoted accountability, and motivated both caregivers and staff to achieve daily KMC targets. Encouraging mothers to actively participate in infant care further strengthened family engagement and aligned care with parent-centred KMC principles.

Environmental modifications complemented these interventions by addressing common barriers to prolonged KMC. Promoting nighttime KMC, providing educational reading materials, and creating a comfortable, private, and home-like environment helped reduce maternal fatigue and overcome challenges related to restricted visiting hours. In addition, systematic documentation of daily KMC duration and routine review during clinical rounds embedded KMC monitoring into standard neonatal care, enhancing compliance and long-term sustainability. By the end of PDSA Cycle 2, the project achieved an average KMC duration of 6.19 hours per day. Although this remains below the WHO recommendation of eight hours daily, it represents a clinically meaningful improvement achieved through low-cost, context-specific interventions using QI methodology. The findings demonstrate that iterative PDSA cycles combining education, workflow modifications, behavioural strategies, environmental support, and continuous monitoring can substantially improve both the early initiation and sustained practice of KMC in resource-limited neonatal settings, as shown in Figure 2. 

A total of 60 mother-infant dyads participated during the 75-day implementation phase of the QI initiative. Baseline demographic and clinical characteristics were evaluated and compared across the baseline period, PDSA Cycle 1, and PDSA Cycle 2. There was a statistically significant difference in the mean value across the three phases (baseline, PDSA 1, and PDSA 2) (F (2, 57) = 12.49, p < 0.001), indicating a significant change over the QI intervention periods. The mean maternal age declined from 34.53 years at baseline to 27.08 years in PDSA Cycle 2, suggesting increased involvement of younger mothers in KMC over the course of the study. The mean gestational age of the neonates increased from 29.92 weeks during the baseline phase to 32.87 weeks in PDSA Cycle 1 and was 31.37 weeks in PDSA Cycle 2. Likewise, the mean birth weight improved from 1,151 g at baseline to 1,285 g and 1,291 g during PDSA Cycles 1 and 2, respectively. With respect to the mode of delivery, the proportion of normal vaginal deliveries rose from 23.08% at baseline to 60.86% in PDSA Cycle 2, while the proportion of lower-segment caesarean section (LSCS) deliveries decreased from 76.92% to 39.14%, as shown in Table 2.

**Table 2:** Comparison of demographic and clinical characteristics across baseline, PDSA 1, and PDSA 2 phases LSCS: lower-segment caesarean section, PDSA: Plan-Do-Study-Act

Characteristic	Baseline (n = 13)	PDSA Cycle 1 (n = 24)	PDSA Cycle 2 (n = 23)	Statistic	p-value
Maternal age, M (SD)	34.53 (3.38)	30.16 (5.66)	27.08 (2.87)	F = 12.49	<0.001
Gestational age, M (SD)	29.92 (2.10)	32.87 (1.80)	31.37 (3.12)	F = 6.50	0.003
Birth weight (g), M (SD)	1151 (252)	1285 (262)	1291 (293)	F = 1.44	0.246
Normal vaginal delivery, n (%)	3 (23.08%)	6 (25.00%)	14 (60.86%)	χ² = 7.25	0.027
LSCS delivery, n (%)	10 (76.92%)	18 (75.00%)	9 (39.14%)	χ² = 7.25	0.027

Maternal age and gestational age showed statistically significant differences across the three phases (p < 0.05), indicating possible case-mix variation over time. Birth weight was comparable across phases. The mode of delivery distribution also differed significantly between phases. These differences were considered potential confounding factors and have been acknowledged as limitations when interpreting the association between QI interventions and KMC outcomes. 

Post-hoc pairwise comparisons were performed after one-way ANOVA. Maternal age differed significantly between baseline and PDSA 2 (p < 0.001) and between PDSA 1 and PDSA 2 (p = 0.012). Gestational age differed significantly between baseline and PDSA1 (p = 0.002). No other pairwise comparisons showed statistically significant differences; during the baseline assessment, delayed initiation and insufficient duration of KMC were recognized as significant gaps in NICU practices. In-depth interviews and focus group discussions conducted with mothers and healthcare personnel identified multiple barriers to effective KMC implementation, including inadequate awareness of KMC benefits and procedures, maternal apprehension about handling low birth weight neonates, increased staff workload, inconsistent counselling practices, interruptions during KMC sessions, and the absence of structured monitoring mechanisms.

To address the identified barriers, sequential PDSA cycles were implemented. In the first cycle, educational interventions were introduced to improve awareness and adherence to KMC practices. Structured presentations and in-service training sessions were conducted for healthcare providers, particularly nursing officers, to standardize KMC practices and strengthen support for mothers initiating KMC. Mothers and family members received daily counselling and health education sessions to encourage participation. A mother support group was established to promote peer learning and encouragement. Additional measures included minimising interruptions during KMC sessions, using validated educational videos, and introducing specially designed gowns to facilitate early and comfortable skin-to-skin contact. After implementation of the first PDSA cycle, the median time for initiation of KMC decreased from 18 days during the baseline phase to 10 days. In addition, the median daily duration of KMC increased from 3.28 hours/day at baseline to 4.08 hours/day, reflecting better adherence to prolonged KMC practices.

During the second PDSA cycle, interventions aimed to sustain and enhance KMC adherence and duration of approximately 6.19 hours, ensuring that each shift included at least two to three hours of engagement by the researchers. Daily KMC hours were documented during morning rounds to identify mothers needing additional support and counselling. The researcher updated KMC records daily, and cases of non-adherence were communicated to treating physicians for reinforcement counselling with family members. A smiley-based KMC monitoring card was introduced to simplify documentation and motivate mothers. Additional measures included establishing a dedicated breast milk expression room, promoting nighttime KMC, and providing reading materials to improve maternal comfort during prolonged KMC sessions.

Following these interventions, the median time to initiate KMC further decreased to day 7 during the second PDSA cycle. The median daily KMC duration increased to 6.19 hours/day, achieving the target for prolonged KMC. Nursing staff ensured two to three hours of supervised KMC during each shift. Overall, successive PDSA cycles demonstrated that structured QI interventions effectively improved both early initiation and prolonged duration of KMC among VLBW neonates in the NICU.

In our study, the median daily KMC duration increased from 3.28 hours at baseline to 6.19 hours by the end of PDSA Cycle 2, representing a clinically meaningful improvement. Although this did not achieve the globally recommended target of eight hours per day, substantial progress was observed. The data points below six hours shown in Figure 2 primarily reflect challenges encountered during the early implementation phase, including shift transitions, significant maternal postpartum pain and fatigue following LSCS deliveries (which accounted for 76.9% of mothers at baseline and 75% during PDSA Cycle 1), and temporary interruptions when mothers left the NICU for medical evaluations.

## Discussion

This QI initiative demonstrated that a structured, multifaceted intervention can substantially improve both the early initiation and duration of KMC among VLBW neonates admitted to the NICU. Following sequential PDSA cycles, the median age of KMC initiation improved from 18 days at baseline to 10 days during PDSA Cycle 1 and further to seven days during PDSA Cycle 2. Similarly, the mean duration of KMC increased from 3.28 hours/day at baseline to 4.08 hours/day and finally to 6.19 hours/day. These findings indicate that systematic interventions targeting institutional, caregiver, and environmental barriers can significantly enhance KMC practices even in resource-constrained settings.

The observed improvements are clinically meaningful because growing evidence suggests that the benefits of KMC are dose-dependent, with earlier initiation and longer duration resulting in greater reductions in neonatal morbidity and mortality. Lawn et al. reported that KMC substantially reduces deaths due to preterm birth complications, particularly when initiated early and continued for prolonged periods [17]. Previous studies have also demonstrated that prolonged skin-to-skin contact improves thermoregulation, cardiorespiratory stability, breastfeeding, weight gain, neurodevelopment, and parent-infant bonding while reducing hypothermia, sepsis, and mortality among preterm infants [1,2,17]. Therefore, improving the quality of KMC implementation has important implications beyond increasing KMC coverage alone.

Fishbone analysis in the present study identified multiple barriers to optimal KMC implementation, including the absence of a written KMC policy, inadequate staff training, maternal anxiety regarding handling fragile neonates, lack of alternative caregivers, interruptions during KMC sessions, insufficient counselling, and infrastructural constraints. Similar barriers have been reported in other low- and middle-income countries (LMICs), where inadequate infrastructure, increased staff workload, sociocultural challenges, and limited institutional support frequently impede KMC implementation [18]. Addressing these barriers through educational interventions, workflow modifications, behavioural strategies, and environmental support resulted in sustained improvements in KMC practices, highlighting the importance of adopting a systems-based approach rather than isolated interventions.

The increase in KMC duration observed in our study is comparable with findings from other QI initiatives conducted in LMIC settings. Joshi et al. reported significant improvements in KMC practices following staff sensitization, caregiver education, and continuous monitoring strategies in a teaching hospital setting [19]. Similarly, multicountry implementation studies have demonstrated that structured interventions, family engagement, and institutional commitment can substantially improve KMC uptake and adherence [18]. The increase in KMC duration from 3.28 to 6.19 hours/day observed in our study is consistent with previous reports showing improvements in daily KMC duration following implementation of targeted QI interventions.

However, despite substantial improvements, the average duration of KMC remained below the WHO recommendation of at least eight hours/day. Similar challenges have been described in other LMIC settings, where maternal fatigue, staffing limitations, competing clinical priorities, and infrastructural constraints hinder the achievement of prolonged KMC targets [18]. Likewise, although the median age of KMC initiation improved to seven days, initiation remained later than ideal recommendations advocating KMC initiation as early as possible after clinical stabilization. These findings emphasize that while significant progress can be achieved through QI interventions, additional efforts are required to overcome persistent barriers and promote continuous KMC practices.

The successful implementation of this project may be attributed to the use of iterative PDSA cycles, which enabled continuous identification of barriers and timely adaptation of interventions. The PDSA methodology has been widely recognized as an effective framework for healthcare QI because it facilitates context-specific testing and refinement of interventions through small, sequential changes [20,21]. In our study, educational sessions, daily counselling, establishment of mother support groups, introduction of monitoring cards, and environmental modifications collectively contributed to improved staff engagement and caregiver participation.

A notable strength of the present study is its focus on both early initiation and prolonged duration of KMC, as most previous studies have primarily concentrated on KMC coverage alone. Furthermore, the use of low-cost, context-specific interventions, including smiley-based monitoring cards, educational videos, and integration of KMC documentation into routine rounds, provides practical strategies that can be replicated in similar resource-limited NICUs. Continuous monitoring and feedback mechanisms embed KMC into routine neonatal care and may contribute to long-term sustainability of the interventions. Previous studies have similarly emphasized the importance of monitoring systems and institutional ownership in sustaining KMC implementation [22,23].

The present study also has several limitations. First, it was conducted in a single tertiary care NICU with a relatively small sample size, which may limit the generalizability of findings to other settings. Second, significant differences in maternal age, gestational age, and mode of delivery were observed across study phases, indicating potential case-mix variations that may have influenced KMC practices. These factors should be considered as potential confounders when interpreting the observed improvements. Third, the study primarily evaluated process indicators such as initiation time and duration of KMC and did not assess long-term neonatal outcomes, including mortality, breastfeeding rates, neurodevelopment, or growth parameters. Finally, the relatively short implementation period limited the assessment of sustainability beyond the study duration.

Despite these limitations, the study demonstrates that substantial improvements in KMC practices can be achieved through structured QI methodologies, as shown in the report from the first global congress [24]. The findings support the integration of educational, behavioural, organizational, and environmental interventions into routine neonatal care to strengthen KMC implementation.

## Conclusions

This QI initiative demonstrated that low-cost, structured, and context-specific interventions can significantly improve the implementation of KMC in a tertiary care NICU. While the temporal association between the QI interventions and enhanced KMC initiation and duration is encouraging, differences in patient characteristics across study phases may have contributed to the observed improvements. Therefore, given these demographic differences, it is difficult to isolate the independent contribution of the interventions and may not be conclusively established from the current analysis. Through sequential PDSA cycles, the median age at initiation of KMC decreased from 18 days to seven days, while the median daily duration of KMC increased from 3.28 hours to 6.19 hours, indicating substantial improvement in adherence to KMC practices. Key interventions, including healthcare staff training, maternal education, structured counselling, family involvement, continuous monitoring, and supportive environmental modifications, contributed to improved acceptance and integration of KMC into routine neonatal care. Addressing barriers, such as inadequate awareness, inconsistent counselling practices, maternal discomfort, and resistance to change, played an important role in achieving these improvements. Although the WHO-recommended target of at least eight hours of daily KMC was not fully achieved, the observed increase to more than six hours/day represented a clinically meaningful and sustainable improvement within a resource-limited setting. The findings suggest that multidisciplinary quality improvement approaches can strengthen KMC implementation and promote better neonatal care practices in NICUs.
